# Response of Yield Parameters of Field Pea (*Pisum sativum* L.) to Different Shading Levels Under Rainfed Conditions

**DOI:** 10.3390/plants15213321

**Published:** 2026-10-27

**Authors:** Sibel İpekeşen, Süreyya Betül Rufaioğlu, Murat Tunç, Levent Yorulmaz, Mihriban Okur, Doğan İpekeşen, Behiye Tuba Biçer

**Affiliations:** 1 Department of Field Crops, Faculty of Agriculture, Dicle University, Diyarbakır 21280, Türkiye; sibel.ipekesen@dicle.edu.tr (S.İ.); levent.yorulmaz@dicle.edu.tr (L.Y.); 2 Department of Soil Science and Plant Nutrition, Faculty of Agriculture, Harran University, Şanlıurfa 63290, Türkiye; sureyyarufaioglu@harran.edu.tr; 3 Department of Field Crops, Faculty of Agriculture, Harran University, Şanlıurfa 63290, Türkiye; murattunc@harran.edu.tr; 4 Department of Agricultural Machinery and Technologies Engineering, Faculty of Agriculture, Dicle University, Diyarbakır 21280, Türkiye; mihriban.okur@dicle.edu.tr; 5 Department of Plant Protection, Faculty of Agriculture, Dicle University, Diyarbakır 21280, Türkiye; dogan21n@gmail.com

**Keywords:** biomass, field pea, *Pisum sativum* L., shade net, shading level, yield components

## Abstract

Reduced irradiance under shade nets may help cool-season legumes cope with the high radiation and heat load of late spring in semi-arid regions, but the response of field pea to graded shading under rainfed conditions is poorly documented. This study evaluated the yield and yield components of two field pea varieties (Araka and Yaprak) grown under full sunlight (control) and 40%, 75% and 90% shading in a two-year (2022 and 2023) rainfed field experiment in Diyarbakır, Türkiye, arranged as a split-plot randomized complete block design with three replications. Year, variety and shading level, and their interactions, significantly affected most traits, so responses were evaluated for each year and variety. Averaged over years and varieties, shading increased plant height by 24–27% relative to full sunlight, and 40% shading increased pod weight (+31%), number of seeds (+30%) and seed weight per plant (+25%). However, these per-plant gains did not translate into higher yield per unit area: biomass yield was highest under full sunlight (3.06 t ha^−1^) and decreased by 16–28% under shading, while seed yield under 40% shading (1.09 t ha^−1^) did not differ from that under full sunlight (1.10 t ha^−1^) and was reduced by 13% and 25% under 75% and 90% shading, respectively. Seed yield responses depended on variety and year: 90% shading reduced seed yield of both varieties in both years (by 9–35%), Araka yielded 19–24% more under 75% shading than under full sunlight in both years, and Yaprak gave its highest seed yield under 40% shading in 2022 (1.23 t ha^−1^) and under full sunlight in 2023 (1.96 t ha^−1^). After the year effect was removed, seed yield was closely correlated with biomass yield (r = 0.91) but not with the per-plant yield components. Heavy shading (90%) should therefore be avoided, whereas light shading (40%) can be applied without a yield penalty on average; the optimum level depends on variety and seasonal conditions.

## 1. Introduction

Global climate change is making it increasingly difficult to meet the nutritional needs of the growing world population. Underdeveloped and developing countries in particular cannot meet essential nutritional requirements such as protein, vitamins and minerals because of cost. Therefore, edible grain legumes such as pea, dry bean, faba bean and lentil are widely cultivated and consumed for their seeds in these countries [1,2].

Field pea (*Pisum sativum* L.) is one of the most popular edible legumes and an important source of protein, vitamins and minerals for human and animal diets among plant-based proteins. Plant-based proteins are preferred in order to minimise threats to the environment and to human and animal health, especially in recent years [3]. Pea is produced in more than 95 countries worldwide, with an annual production of around 15.7 million t [4]. Türkiye produces only 3.8 thousand t, which is considerably lower than in other producing countries [5]. Pea is a cost-effective and high-yielding protein source among legumes and its production should be encouraged in Türkiye. However, pea production is restricted by various factors, particularly high temperatures during the reproductive period [6,7].

Climate change may reduce pea yield through high temperatures, soil moisture deficit, drought and heat stress, particularly in arid and semi-arid zones [7]. Pea is a cool-season crop, and temperatures above approximately 25 °C during flowering and pod filling reduce seed set, seed number and seed weight [6,7,8]. In such environments, the crop is frequently exposed to high irradiance and high temperature simultaneously during the reproductive period, and reducing the incident radiation load is one of the management options proposed to mitigate these combined stresses [9]. However, sunlight is also the primary resource for crop growth: yield depends on how much radiation the canopy intercepts and how efficiently it converts that radiation into photosynthates [10]. Any reduction in irradiance therefore involves a trade-off between alleviating excess radiation and heat load and limiting the energy available for photosynthesis.

Plants respond to reduced light through two broad strategies. Shade-avoiding plants elongate stems and petioles and allocate more resources to height growth, often at the expense of branching and reproductive allocation, whereas shade-tolerant plants adjust leaf morphology and physiology to maintain carbon gain under low light [11,12,13]. Under shading, light absorption, chlorophyll content, photosynthetic rate and the radiation balance of the canopy change in ways that depend on species, shade intensity and duration [14]. These physiological variables were not measured in the present study; they are mentioned here because they provide the framework within which the measured morphological and yield traits are interpreted. The effects of shade nets also depend on shade density, weave type and colour [15,16,17,18,19]. In legumes, moderate shading has been reported to maintain or even increase biomass in shade-tolerant species, whereas heavy shading generally reduces pod number and yield [14,20,21,22]. In faba bean, shading delayed physiological maturity and extended the seed-filling period, resulting in higher seed weight and grain yield [23], whereas in soybean, shading reduced pod production and pod set [21].

Information on field pea is limited. Previous studies have examined the effect of light intensity on pea microgreens under controlled conditions [2], the interaction between light intensity and nitrogen on pea growth [24] and the effect of shading applied at different developmental stages on leaf pea [25]. Field studies comparing several shading levels on field pea varieties over more than one season under rainfed semi-arid conditions are scarce. In particular, it is not known whether the increases in individual-plant traits that are often observed under moderate shading translate into higher seed yield per unit area, and whether the response is consistent across varieties and years. This information is needed to judge whether shade nets, which are widely used to protect horticultural crops from excessive radiation [15,17], could also be a practical option for field pea in regions where late-spring heat is expected to intensify.

Seed yield of grain legumes, including pea, is determined by the number of plants per unit area, the number of pods per plant, the number of seeds per pod and individual seed weight, and these components are set sequentially during the reproductive period [26,27]. In the present study, plant height and plant biomass were measured to describe the shade-avoidance and assimilate-supply responses of the plant; pod length and pod width to describe the size of individual pods; and the number of pods, pod weight, number of seeds and seed weight per plant to describe the reproductive allocation of individual plants. Biomass yield and seed yield per unit area integrate these individual-plant responses with stand-level effects. Measuring both levels allows evaluation of whether changes in individual-plant traits under shading are reflected in productivity per unit area.

The aim of this study was to determine the effects of three shading levels (40%, 75% and 90%), compared with full sunlight, on plant growth, yield components and seed yield of two field pea varieties grown under rainfed conditions over two growing seasons. We hypothesised that (i) increasing shading would progressively limit light availability and reduce biomass and seed yield per unit area; (ii) light shading could improve individual-plant yield components by reducing the radiation and heat load during the reproductive period; and (iii) the magnitude and direction of these responses would differ between varieties and years.

## 2. Results

Analysis of variance showed that year (Y), variety (V), shading level (S) and most of their interactions significantly affected the investigated traits (Table 1 and Table 2). The Y × V × S interaction was significant for all traits, indicating that the varietal response to shading differed between the two growing seasons. Therefore, Y × V × S means are presented for each year in the figures accompanying each trait (Section 2.1, Section 2.2, Section 2.3, Section 2.4, Section 2.5, Section 2.6, Section 2.7, Section 2.8 and Section 2.9), and the means across years (V × S) and main-effect means are given in Table 1 and Table 2. Growing conditions differed markedly between the seasons (Section 4.2): the 2023 season received more rainfall from January to June (371.5 vs. 247.7 mm) and had a lower maximum temperature in May (32.5 vs. 37.8 °C). Plant biomass, the number of pods, pod weight, number of seeds and seed weight per plant were 2.0–3.4 times higher in 2023 than in 2022, and seed yield was 46% higher (1.18 vs. 0.81 t ha^−1^).

### 2.1. Plant Height

**Figure 1:**
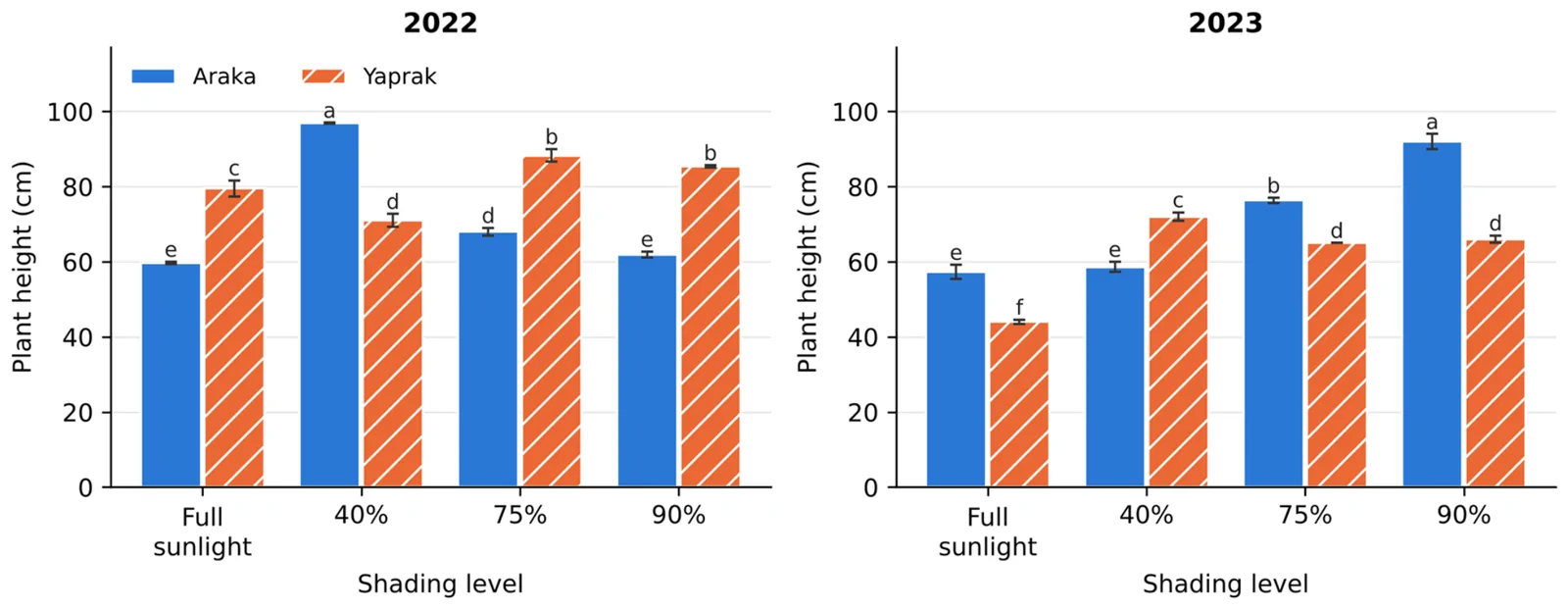
Plant height of two field pea varieties (Araka and Yaprak) grown under full sunlight and 40%, 75% and 90% shading in 2022 and 2023. Bars show means ± standard error of three replications of each year × variety × shading combination. Lowercase letters above the bars indicate the results of Fisher’s least significant difference (LSD) test at *p* ≤ 0.05, based on the error b mean square of the combined analysis of variance (Y × V × S interaction; LSD = 3.37 cm). Comparisons were made separately within each year among the eight variety × shading combinations; bars sharing the same letter within a year do not differ significantly, and letters should not be compared between years.

Plant height was significantly affected by Y, S and all interactions, whereas the two varieties did not differ when averaged over years and shading levels (71.35 vs. 71.40 cm; Table 1). In both years, plants grown under full sunlight were the shortest or did not differ from the shortest, except for Yaprak in 2022 (Figure 1). The shading level that produced the tallest plants varied with year and variety: in 2022, Araka was tallest under 40% shading (96.86 cm) and Yaprak under 75% and 90% shading (88.31 and 85.39 cm), whereas in 2023, Araka was tallest under 90% shading (92.00 cm) and Yaprak under 40% shading (72.00 cm). Averaged over years and varieties, all shading levels increased plant height relative to full sunlight (60.13 cm), by 23.7–26.9%, with the greatest value under 90% shading (76.33 cm; Table 1).

### 2.2. Plant Biomass

**Figure 2:**
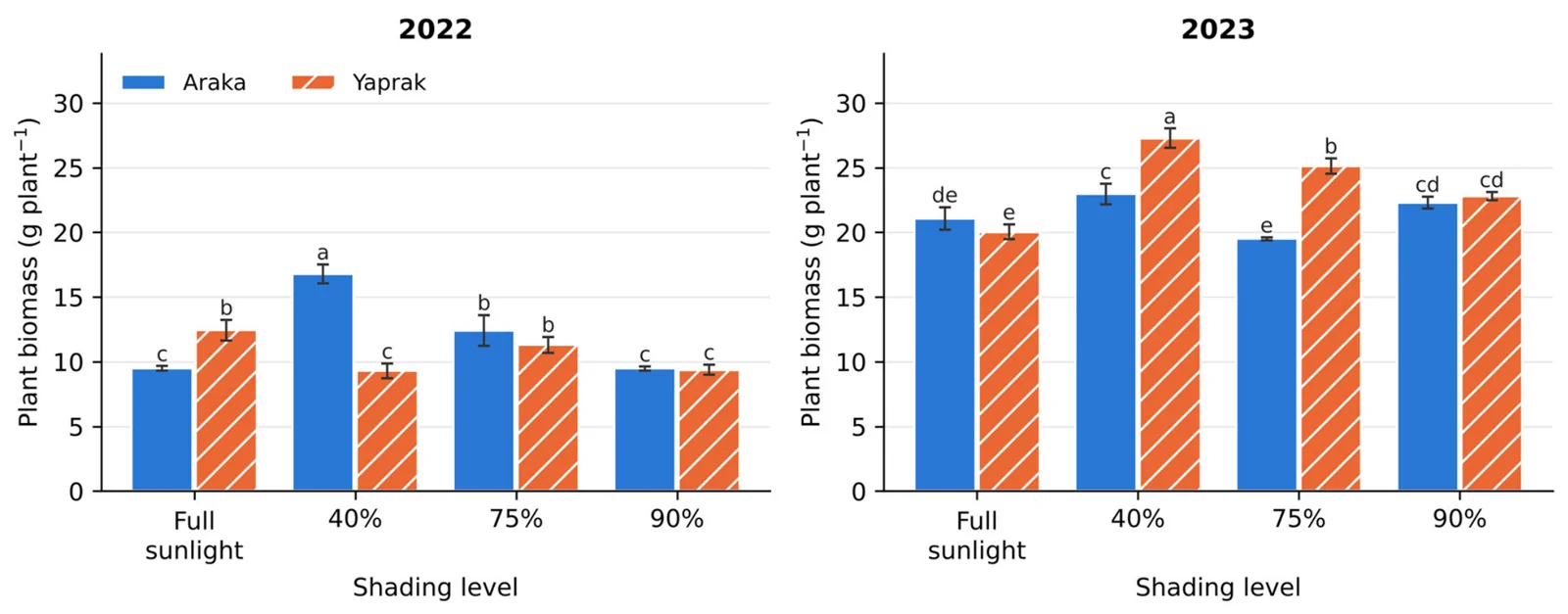
Plant biomass of two field pea varieties (Araka and Yaprak) grown under full sunlight and 40%, 75% and 90% shading in 2022 and 2023. Bars show means ± standard error of three replications of each year × variety × shading combination. Lowercase letters above the bars indicate the results of Fisher’s least significant difference (LSD) test at *p* ≤ 0.05, based on the error b mean square of the combined analysis of variance (Y × V × S interaction; LSD = 1.81 g). Comparisons were made separately within each year among the eight variety × shading combinations; bars sharing the same letter within a year do not differ significantly, and letters should not be compared between years.

Plant biomass was affected by Y, Y × V, S, Y × S, V × S and Y × V × S, but not by variety (Table 1). It was twice as high in 2023 as in 2022 (22.63 vs. 11.33 g), whereas Araka and Yaprak did not differ (16.75 vs. 17.21 g). Averaged over years and varieties, 40% shading gave the highest plant biomass (19.08 g; +21.1% relative to full sunlight), followed by 75% shading (17.09 g; +8.5%), whereas 90% shading (15.99 g; +1.5%) did not differ from full sunlight (15.76 g). The variety × shading pattern differed between years (Figure 2): 40% shading markedly increased plant biomass of Araka in 2022 (16.80 vs. 9.49 g; +77%) and of Yaprak in 2023 (27.27 vs. 20.03 g; +36%) and, to a lesser extent, of Araka in 2023 (22.97 vs. 21.07 g; +9%), but reduced that of Yaprak in 2022 (9.29 vs. 12.44 g; −25%). Under 90% shading, plant biomass of Yaprak was lower than under full sunlight in 2022 (−25%) but higher in 2023 (+14%), whereas that of Araka did not differ from full sunlight in either year.

### 2.3. Pod Length and Pod Width

**Figure 3:**
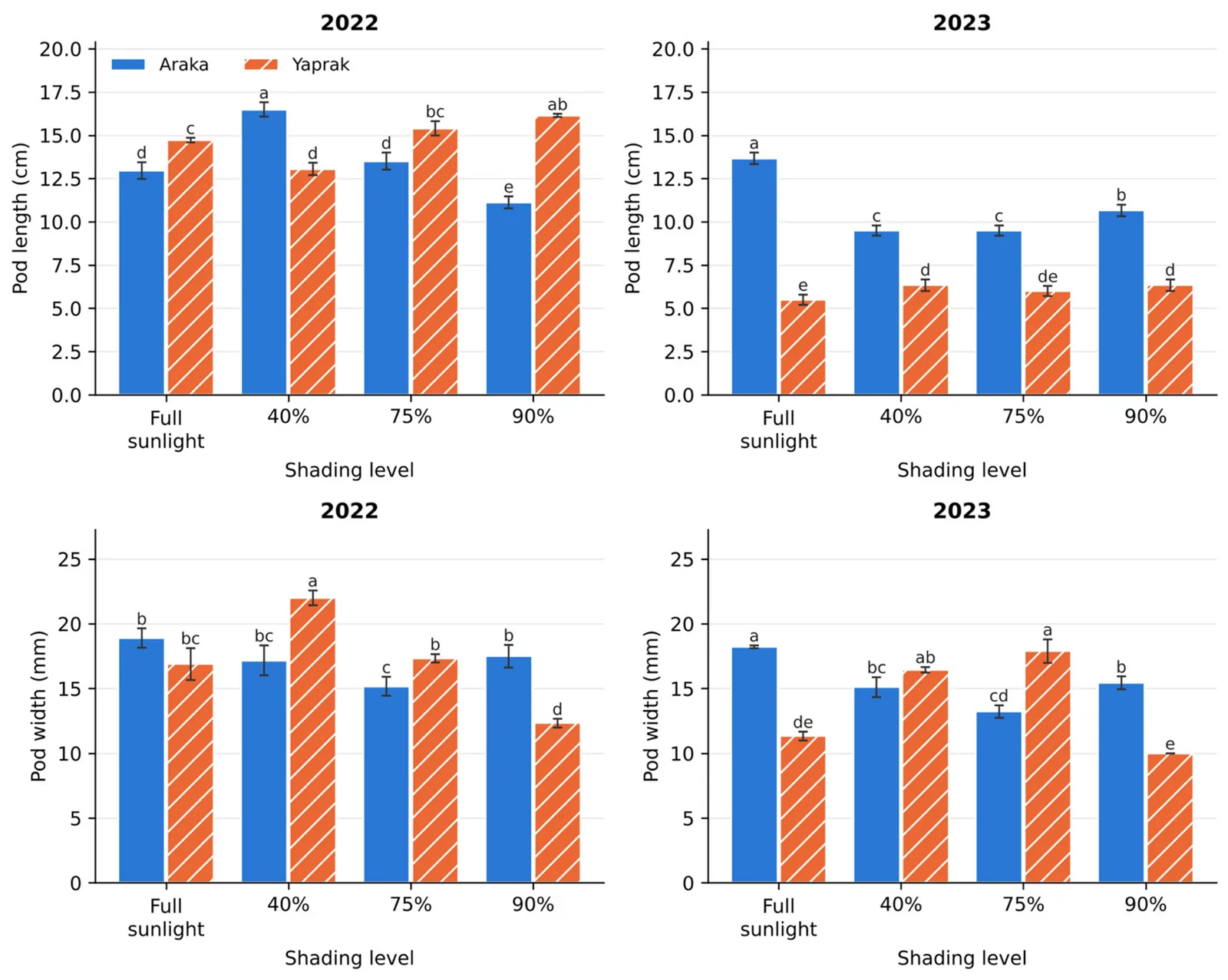
Pod length (**upper panels**) and pod width (**lower panels**) of two field pea varieties (Araka and Yaprak) grown under full sunlight and 40%, 75% and 90% shading in 2022 and 2023. Bars show means ± standard error of three replications of each year × variety × shading combination. Lowercase letters above the bars indicate the results of Fisher’s least significant difference (LSD) test at *p* ≤ 0.05, based on the error b mean square of the combined analysis of variance (Y × V × S interaction; LSD = 0.82 cm for pod length and 2.09 mm for pod width). Comparisons were made separately within each year among the eight variety × shading combinations; bars sharing the same letter within a year do not differ significantly, and letters should not be compared between years.

Pod length was affected by all sources of variation (Table 1). Pods were much longer in 2022 than in 2023 (14.17 vs. 8.44 cm), and Araka had longer pods than Yaprak (12.18 vs. 10.43 cm). The shading effect averaged over years and varieties was small: pods under full sunlight (11.71 cm) were 5% longer than those under 75% and 90% shading. In 2023, shading reduced the pod length of Araka by 22–31% compared with full sunlight (13.67 cm), whereas in 2022, the longest pods were obtained under 40% shading in Araka (16.49 cm) and under 90% shading in Yaprak (16.14 cm) (Figure 3). Pod width was affected by Y, S, Y × S, V × S and Y × V × S, but not by variety. Averaged over years and varieties, 40% shading gave the widest pods (17.68 mm; +8.2%) and 90% shading the narrowest (13.82 mm; −15.4%). In Araka, the widest pods were obtained under full sunlight (18.90 mm in 2022 and 18.22 mm in 2023), whereas in Yaprak, the widest pods were obtained under 40% shading in 2022 (22.00 mm) and under 40–75% shading in 2023 (16.44–17.89 mm), and the narrowest pods under 90% shading in both years (12.33 and 10.00 mm) (Figure 3).

### 2.4. Number of Pods per Plant

**Figure 4:**
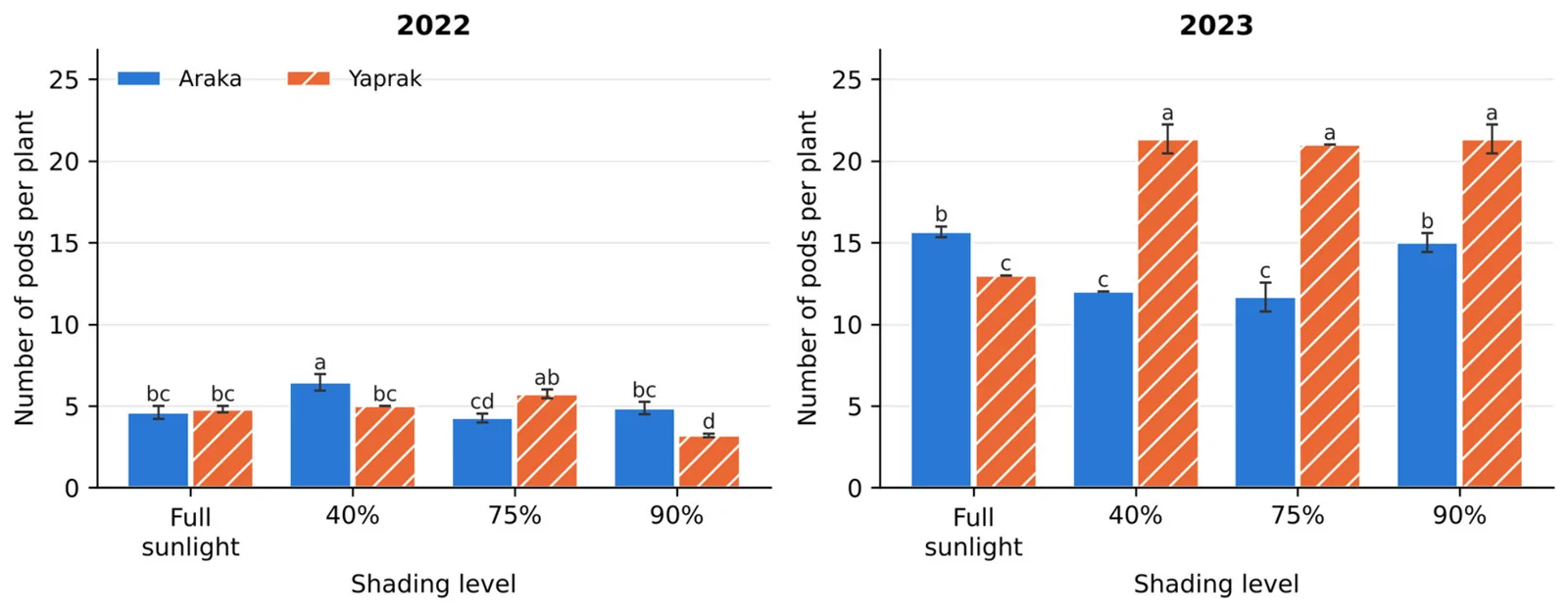
Number of pods per plant of two field pea varieties (Araka and Yaprak) grown under full sunlight and 40%, 75% and 90% shading in 2022 and 2023. Bars show means ± standard error of three replications of each year × variety × shading combination. Lowercase letters above the bars indicate the results of Fisher’s least significant difference (LSD) test at *p* ≤ 0.05, based on the error b mean square of the combined analysis of variance (Y × V × S interaction; LSD = 1.37). Comparisons were made separately within each year among the eight variety × shading combinations; bars sharing the same letter within a year do not differ significantly, and letters should not be compared between years.

The number of pods per plant was affected by all sources of variation (Table 1). It was 3.4 times higher in 2023 than in 2022 (16.38 vs. 4.86) and higher in Yaprak than in Araka (11.93 vs. 9.31). Averaged over years and varieties, all shading levels increased the number of pods per plant by 12–18% compared with full sunlight (9.52). This main effect, however, was mainly due to Yaprak in 2023, in which all shading levels increased the number of pods per plant by 62–64% (Figure 4). In Araka in 2023, 40% and 75% shading reduced the number of pods per plant by 23–26%, whereas in 2022, the highest value was obtained for Araka under 40% shading (6.43) and the lowest for Yaprak under 90% shading (3.20).

### 2.5. Pod Weight per Plant

**Figure 5:**
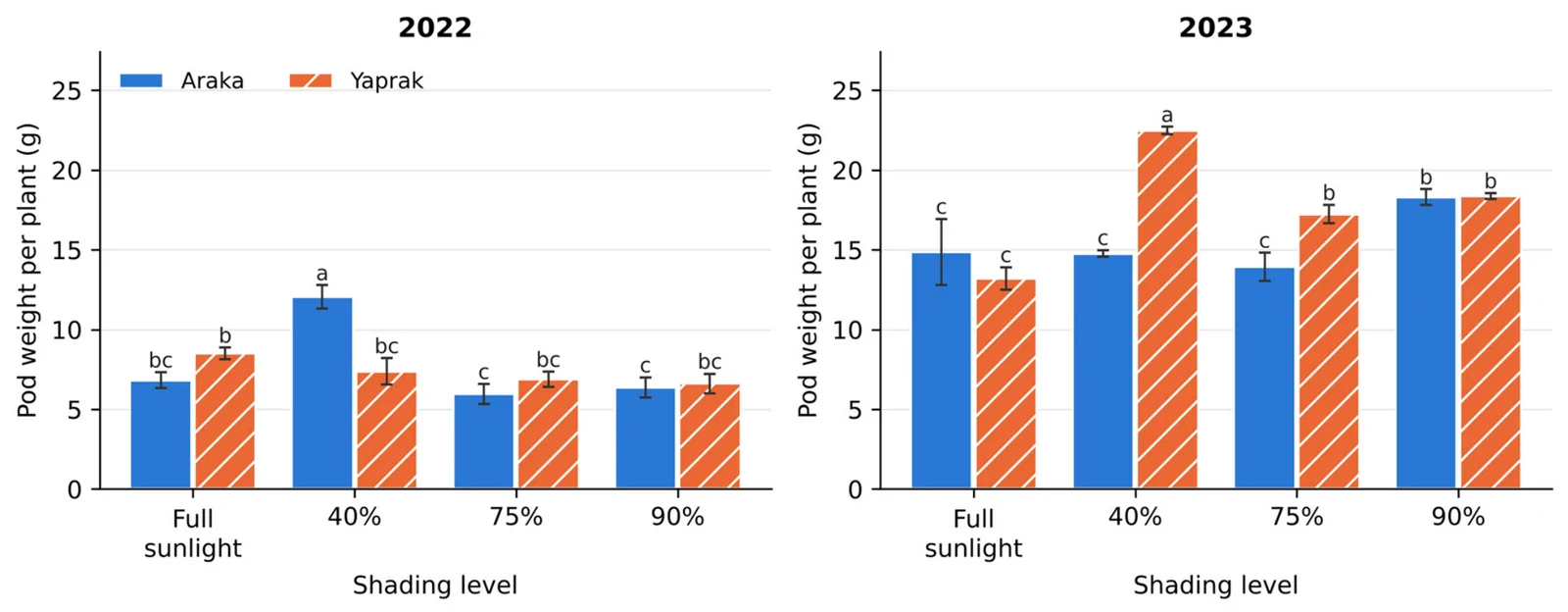
Pod weight per plant of two field pea varieties (Araka and Yaprak) grown under full sunlight and 40%, 75% and 90% shading in 2022 and 2023. Bars show means ± standard error of three replications of each year × variety × shading combination. Lowercase letters above the bars indicate the results of Fisher’s least significant difference (LSD) test at *p* ≤ 0.05, based on the error b mean square of the combined analysis of variance (Y × V × S interaction; LSD = 1.98 g). Comparisons were made separately within each year among the eight variety × shading combinations; bars sharing the same letter within a year do not differ significantly, and letters should not be compared between years.

Pod weight per plant was affected by Y, Y × V, S, Y × S and Y × V × S, but not by variety or V × S (Table 2). Averaged over years and varieties, 40% shading gave the highest pod weight per plant (14.17 g; +30.5% relative to full sunlight), followed by 90% shading (12.41 g; +14.4%), whereas 75% shading (11.00 g) did not differ from full sunlight (10.85 g). The year-specific means (Figure 5) show that the increase under 40% shading occurred in Araka in 2022 (12.05 vs. 6.83 g; +76%) and in Yaprak in 2023 (22.47 vs. 13.20 g; +70%), whereas the increase under 90% shading was restricted to 2023 (Araka, 18.30 vs. 14.87 g; Yaprak, 18.37 vs. 13.20 g). In Yaprak in 2022 and in Araka in 2023, 40% shading did not increase pod weight per plant.

### 2.6. Number of Seeds per Plant

**Figure 6:**
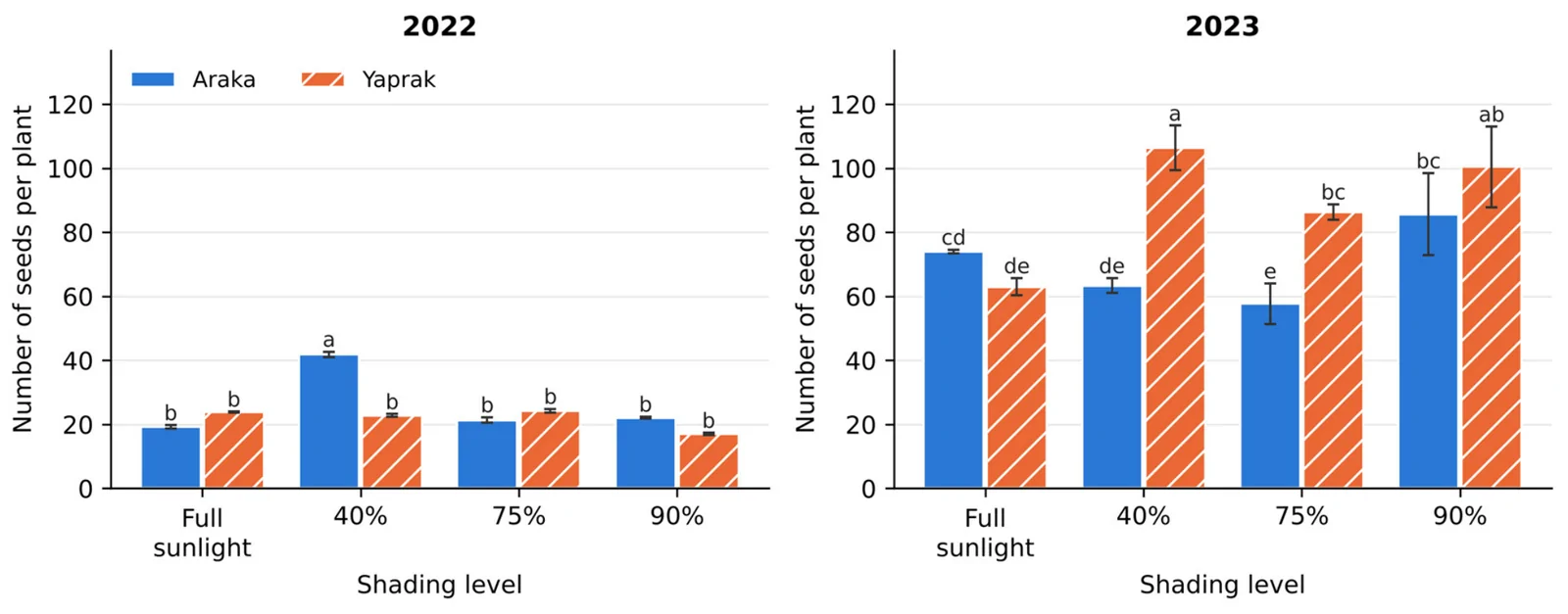
Number of seeds per plant of two field pea varieties (Araka and Yaprak) grown under full sunlight and 40%, 75% and 90% shading in 2022 and 2023. Bars show means ± standard error of three replications of each year × variety × shading combination. Lowercase letters above the bars indicate the results of Fisher’s least significant difference (LSD) test at *p* ≤ 0.05, based on the error b mean square of the combined analysis of variance (Y × V × S interaction; LSD = 15.44). Comparisons were made separately within each year among the eight variety × shading combinations; bars sharing the same letter within a year do not differ significantly, and letters should not be compared between years.

The number of seeds per plant was affected by Y, V, Y × V, S, Y × S and Y × V × S, whereas the V × S interaction was not significant (Table 2). Averaged over years and varieties, 40% and 90% shading increased the number of seeds per plant by 30.1% and 25.1%, respectively, compared with full sunlight (45.02). The year-specific means (Figure 6) show that this increase occurred in Araka under 40% shading in 2022 (41.87 vs. 19.23; +118%) and in Yaprak under all shading levels in 2023 (+37 to +69%), whereas shading did not increase the number of seeds per plant in Yaprak in 2022 or in Araka in 2023.

### 2.7. Seed Weight per Plant

**Figure 7:**
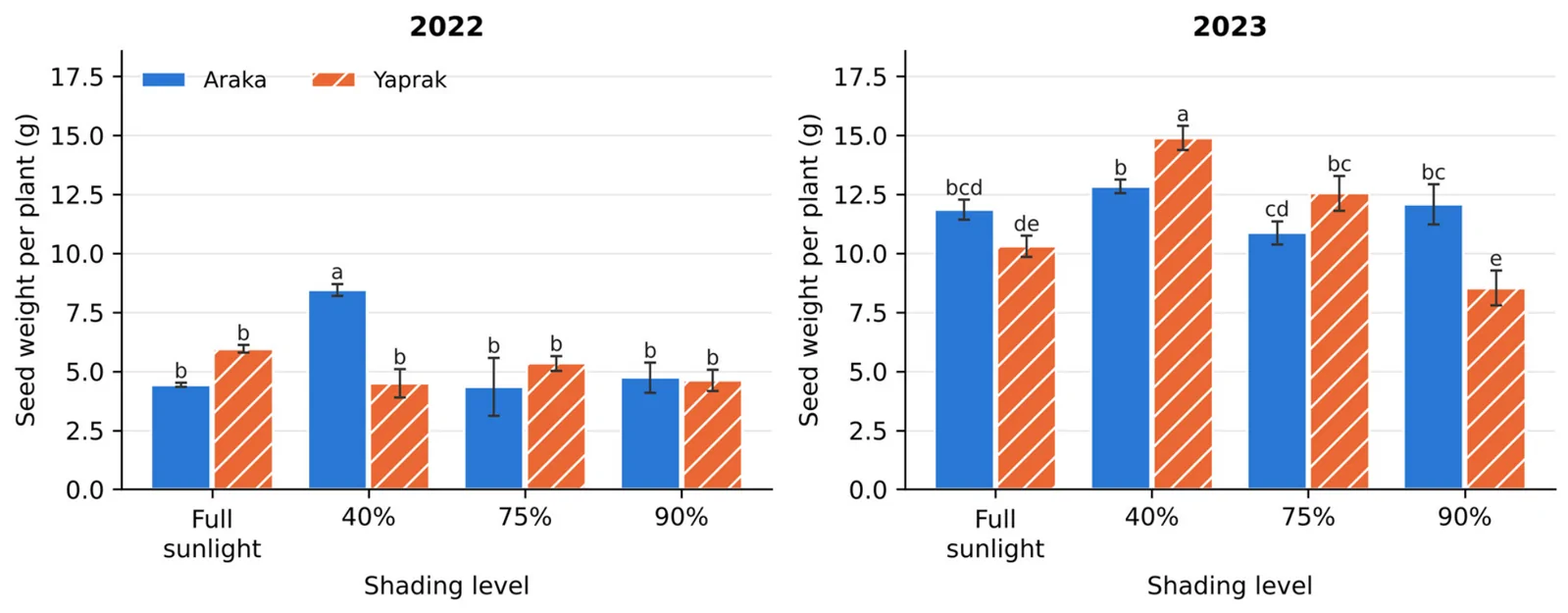
Seed weight per plant of two field pea varieties (Araka and Yaprak) grown under full sunlight and 40%, 75% and 90% shading in 2022 and 2023. Bars show means ± standard error of three replications of each year × variety × shading combination. Lowercase letters above the bars indicate the results of Fisher’s least significant difference (LSD) test at *p* ≤ 0.05, based on the error b mean square of the combined analysis of variance (Y × V × S interaction; LSD = 1.79 g). Comparisons were made separately within each year among the eight variety × shading combinations; bars sharing the same letter within a year do not differ significantly, and letters should not be compared between years.

Seed weight per plant was affected by Y, S, V × S and Y × V × S, but not by variety, Y × V or Y × S (Table 2). Averaged over years and varieties, seed weight per plant was highest under 40% shading (10.17 g; +25.1% relative to full sunlight), whereas 75% and 90% shading did not differ from full sunlight (8.13 g). As for seed number, the increase under 40% shading occurred in Araka in 2022 (8.44 vs. 4.43 g) and in Yaprak in 2023 (14.89 vs. 10.29 g); in Yaprak in 2023, 75% shading also increased seed weight per plant (12.54 g). Shading levels did not differ for Yaprak in 2022 and did not differ from full sunlight for Araka in 2023 (Figure 7).

### 2.8. Biomass Yield

**Figure 8:**
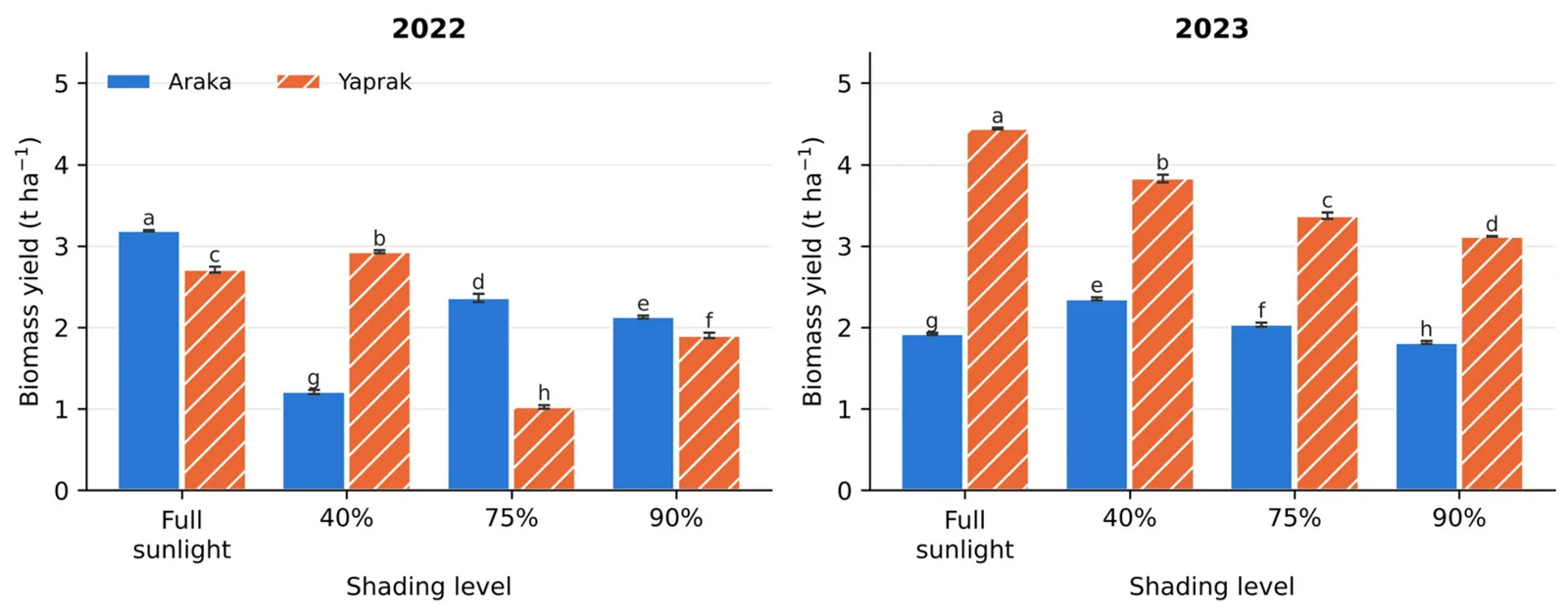
Biomass yield of two field pea varieties (Araka and Yaprak) grown under full sunlight and 40%, 75% and 90% shading in 2022 and 2023. Bars show means ± standard error of three replications of each year × variety × shading combination. Lowercase letters above the bars indicate the results of Fisher’s least significant difference (LSD) test at *p* ≤ 0.05, based on the error b mean square of the combined analysis of variance (Y × V × S interaction; LSD = 0.078 t ha^−1^). Comparisons were made separately within each year among the eight variety × shading combinations; bars sharing the same letter within a year do not differ significantly, and letters should not be compared between years.

Biomass yield was affected by all sources of variation (Table 2). It was higher in 2023 than in 2022 (2.86 vs. 2.18 t ha^−1^) and higher in Yaprak than in Araka (2.92 vs. 2.13 t ha^−1^). Averaged over years and varieties, full sunlight gave the highest biomass yield (3.06 t ha^−1^), and 40%, 90% and 75% shading reduced it by 15.8%, 26.9% and 28.3%, respectively. In Yaprak in 2023, biomass yield decreased progressively with increasing shading, from 4.44 t ha^−1^ under full sunlight to 3.12 t ha^−1^ under 90% shading (−30%). The response in the other year × variety combinations was less regular: in 2022, the highest biomass yield was obtained under full sunlight in Araka (3.19 t ha^−1^) and under 40% shading in Yaprak (2.93 t ha^−1^), and in 2023, Araka produced its highest biomass yield under 40% shading (2.35 t ha^−1^) (Figure 8).

### 2.9. Seed Yield

Seed yield was affected by all sources of variation (Table 2). It was 46% higher in 2023 than in 2022 (1.18 vs. 0.81 t ha^−1^) and 65% higher in Yaprak than in Araka (1.24 vs. 0.75 t ha^−1^). Averaged over years and varieties, seed yield under 40% shading (1.09 t ha^−1^) did not differ from that under full sunlight (1.10 t ha^−1^), whereas 75% and 90% shading reduced seed yield by 13.0% and 25.1%, respectively (Table 2).

**Figure 9:**
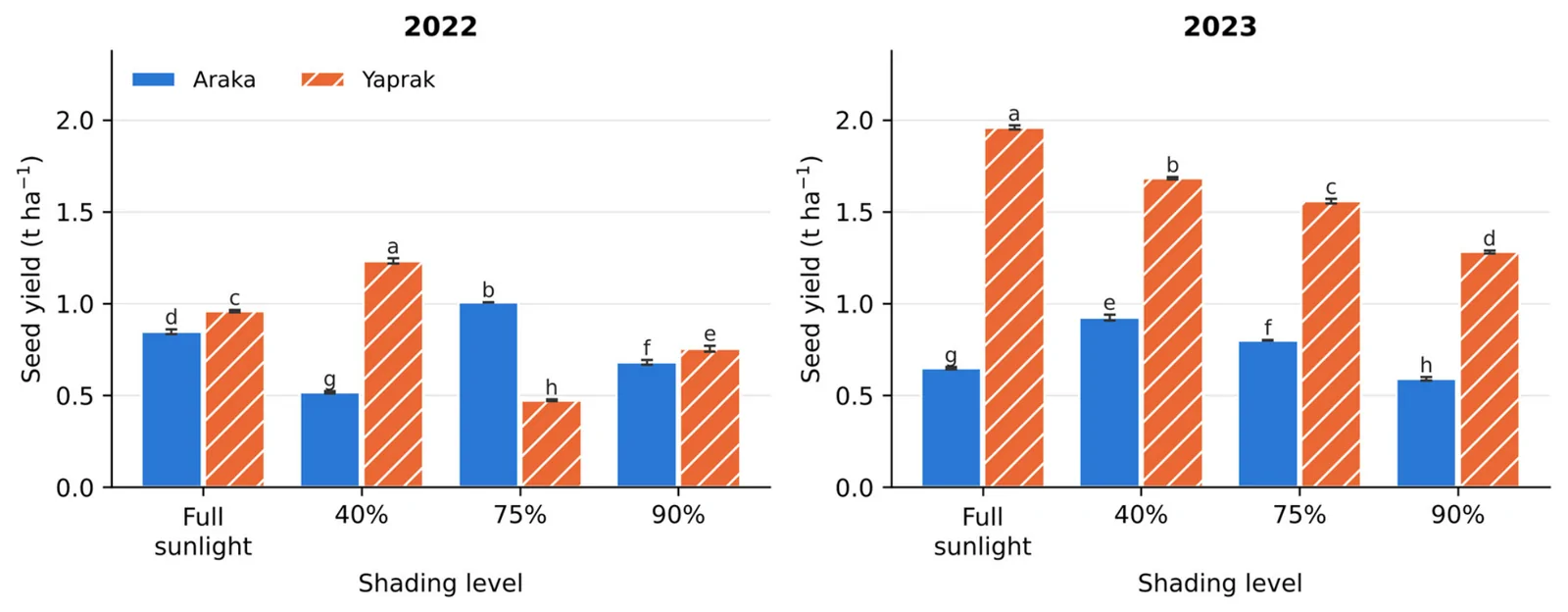
Seed yield of two field pea varieties (Araka and Yaprak) grown under full sunlight and 40%, 75% and 90% shading in 2022 and 2023. Bars show means ± standard error of three replications of each year × variety × shading combination. Lowercase letters above the bars indicate the results of Fisher’s least significant difference (LSD) test at *p* ≤ 0.05, based on the error b mean square of the combined analysis of variance (Y × V × S interaction; LSD = 0.031 t ha^−1^). Comparisons were made separately within each year among the eight variety × shading combinations; bars sharing the same letter within a year do not differ significantly, and letters should not be compared between years.

Three features of the seed yield response were consistent across years (Figure 9). First, 90% shading reduced seed yield of both varieties in both years compared with full sunlight (Araka, −20% and −9%; Yaprak, −21% and −35% in 2022 and 2023, respectively). Second, Araka produced more seed under 75% shading than under full sunlight in both years (1.01 vs. 0.85 t ha^−1^ in 2022 and 0.80 vs. 0.65 t ha^−1^ in 2023). Third, Yaprak yielded less under 75% shading than under full sunlight in both years (−51% and −20%). In contrast, the response of Yaprak to 40% shading differed between years: it produced the highest seed yield of the experiment in 2022 under 40% shading (1.23 t ha^−1^; +28% relative to full sunlight), but in 2023, its highest yield was obtained under full sunlight (1.96 t ha^−1^), and yield decreased progressively with increasing shading. Similarly, Araka gave its highest seed yield under 75% shading in 2022 and under 40% shading in 2023 (0.92 t ha^−1^).

### 2.10. Principal Component and Correlation Analyses

PCA and correlation analyses were performed to examine the relationships among the investigated traits and the distribution of the year × variety × shading observations. Because the year effect was very large for most traits (Table 1 and Table 2), pooling the raw data of the two years would produce correlations that mainly reflect the difference between seasons rather than relationships among traits within a season. Therefore, each variable was standardised within each year before analysis (Section 4.5). The data were suitable for PCA (Kaiser–Meyer–Olkin measure = 0.72; Bartlett’s test of sphericity χ^2^ = 342.3, df = 45, *p* < 0.001).

**Figure 10:**
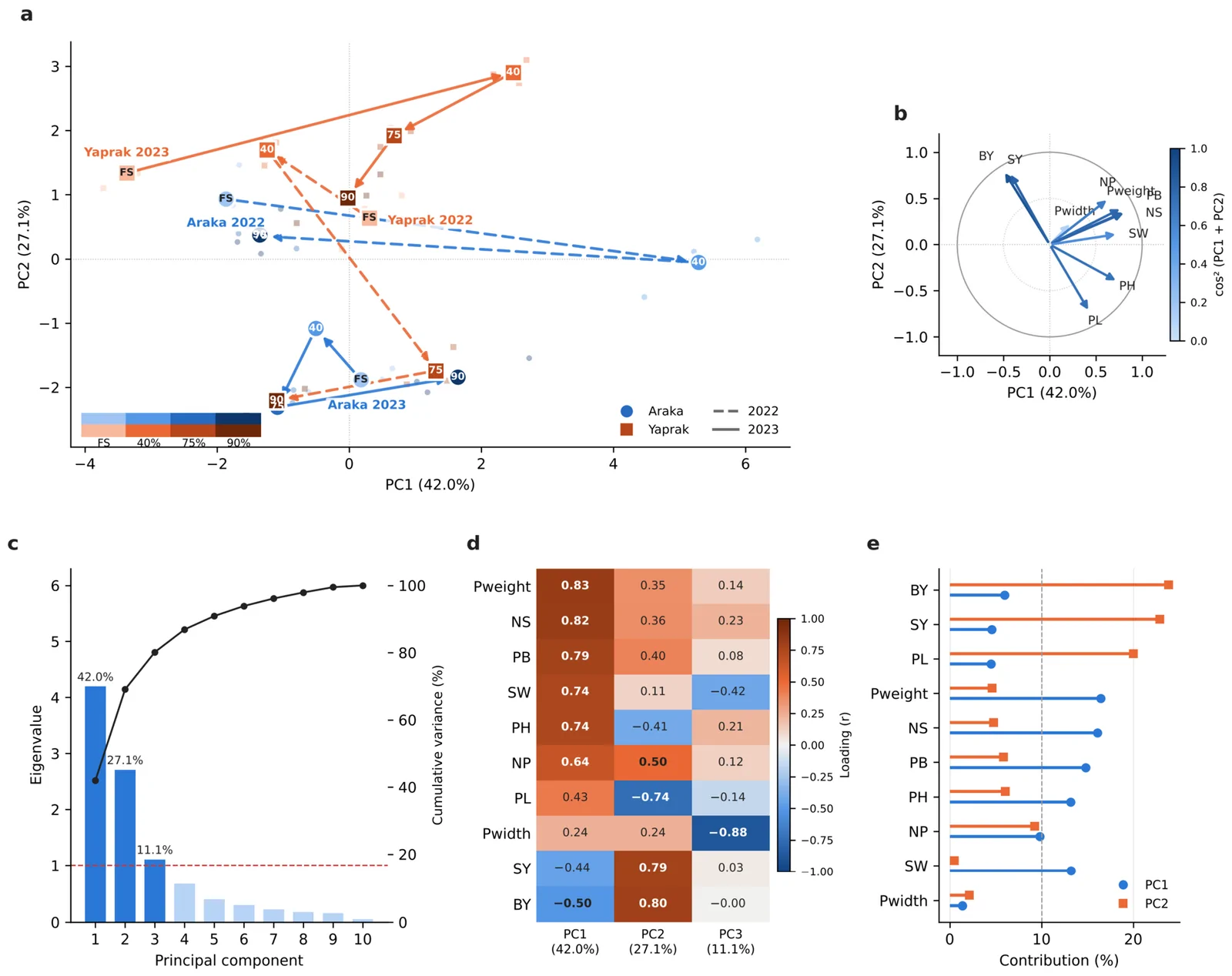
PCA of the investigated traits of two field pea varieties under four shading levels in 2022 and 2023, based on variables standardised within each year. (**a**) Shading response trajectories: large symbols are year × variety × shading centroids, labelled with the shading level and connected by arrows from full sunlight (FS) to 90%; small symbols are individual plots. Circles, Araka; squares, Yaprak; dashed lines, 2022; solid lines, 2023; darker colours indicate higher shading (key at lower left). (**b**) Correlation circle; arrow colour shows the quality of representation (cos^2^). (**c**) Scree plot; dark bars mark components with eigenvalue > 1, and the dashed line marks the Kaiser criterion (eigenvalue = 1). (**d**) Loadings on the first three components; |loading| ≥ 0.50 in bold. (**e**) Contributions of the variables to PC1 and PC2; the dashed line marks the expected average contribution (10%).

The first two principal components explained 69.1% of the total variation (PC1, 42.0%; PC2, 27.1%), and the third component explained a further 11.1% (Figure 10). PC1 was defined mainly by the per-plant traits, namely pod weight (Pweight), number of seeds (NS) and plant biomass (PB) per plant, together with plant height (PH) and seed weight per plant (SW), all of which had positive loadings (0.74–0.83). PC2 was defined mainly by biomass yield (BY, loading 0.80) and seed yield (SY, 0.79), which were positioned opposite to pod length (PL, −0.74) and, to a lesser extent, plant height. The nearly orthogonal positions of the per-plant yield components and the per-area yield traits indicate that these two groups of traits varied largely independently. The shading-response trajectories (Figure 10a) show that the response to shading was not a uniform shift along one axis. Light shading (40%) moved Araka in 2022 and Yaprak in 2023 strongly towards the positive end of PC1 (higher per-plant yield components), but only Yaprak in 2023 remained in the high-PC2 region associated with high biomass and seed yield; Araka under 40% shading in 2022 combined high per-plant yield components with a low seed yield (0.52 t ha^−1^). Higher shading levels (75% and 90%) generally moved the centroids back towards lower PC1 and/or PC2 values, with the largest decline along PC2 for Yaprak in 2022. The four year × variety trajectories differed in direction, which visualises the significant Y × V × S interaction, and the plots of the four shading levels overlapped considerably, so the PCA did not separate the shading levels into distinct groups.

**Figure 11:**
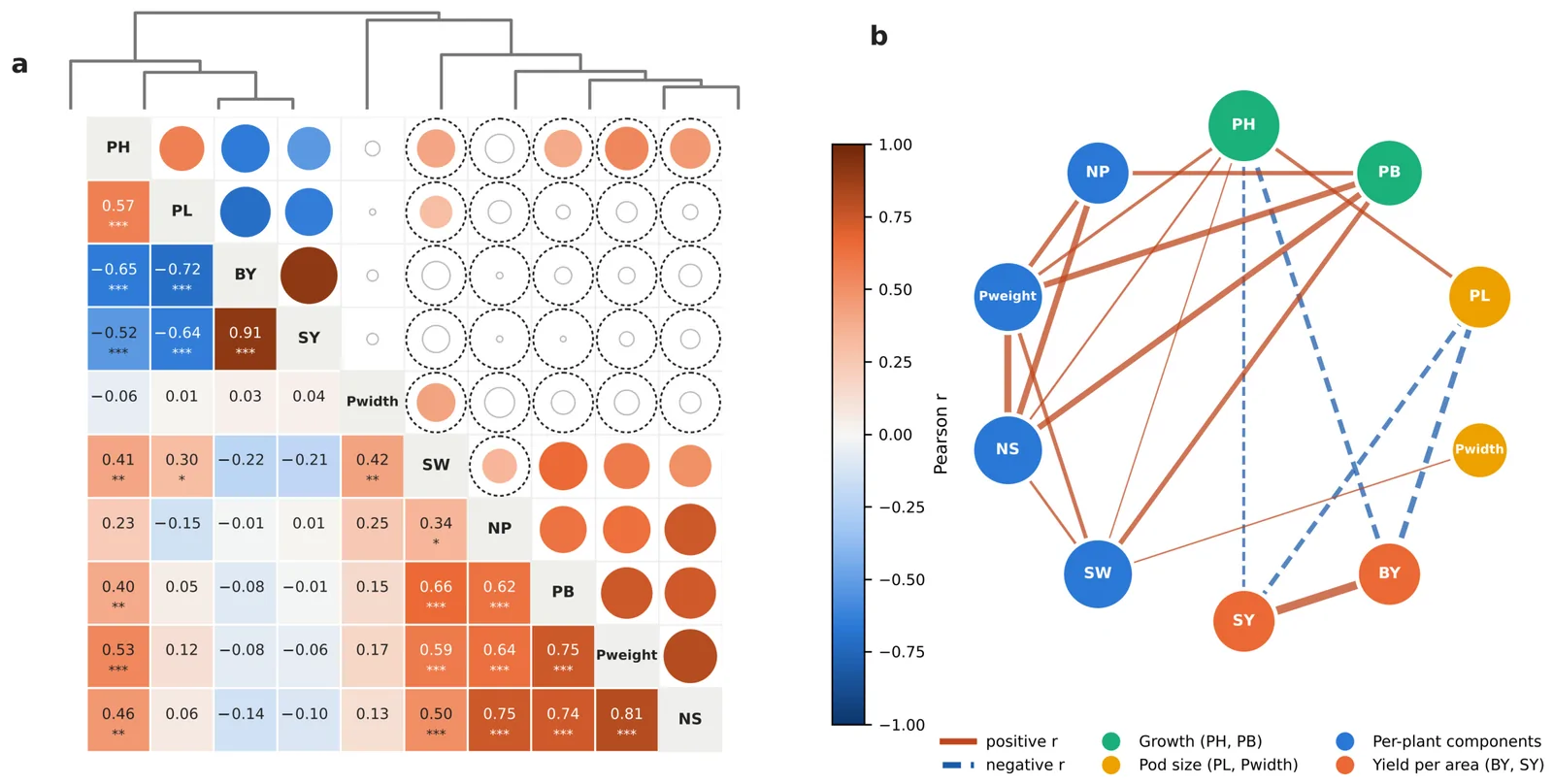
Relationships among the investigated traits of field pea after removal of the year effect. (**a**) Clustered correlogram: variables are ordered by hierarchical clustering (average linkage, distance 1 − |r|); the lower triangle shows Pearson correlation coefficients (*, ** and ***, significant at *p* < 0.05, *p* < 0.01 and *p* < 0.001, respectively), and the upper triangle shows circles with area proportional to |r| (filled, significant; open, not significant). Dashed rings mark pairs for which the correlation calculated from the pooled two-year data differs from the within-year correlation by ≥0.40, i.e., associations driven by the year effect. (**b**) Network of significant correlations (|r| ≥ 0.40, *p* < 0.01): solid red and dashed blue edges indicate positive and negative correlations, edge width is proportional to |r|, node size to the number of significant links and node colour to the trait group.

Within years, seed yield was very strongly and positively correlated with biomass yield (r = 0.91, *p* < 0.001) and negatively correlated with plant height (r = −0.52, *p* < 0.001) and pod length (r = −0.64, *p* < 0.001) (Figure 11). The per-plant yield components were positively intercorrelated (r = 0.34–0.81 among NP, Pweight, NS and SW; *p* < 0.05) and positively correlated with plant biomass (r = 0.62–0.75, *p* < 0.001), but none of them was significantly correlated with seed yield per unit area (r = −0.21 to 0.01). When the raw data of the two years were pooled without removing the year effect, seed yield appeared to be positively correlated with NP, Pweight, NS and SW (r = 0.34–0.52, *p* < 0.05); these associations arose because both the per-plant components and seed yield were higher in 2023 than in 2022, not from a relationship within seasons. Hierarchical clustering grouped the traits into two main clusters (Figure 11a): one containing plant height, pod length, biomass yield and seed yield, connected by strong negative correlations of plant height and pod length with the two yield traits, and one containing pod width, plant biomass and the per-plant yield components. In the correlation network (Figure 11b), seed yield and biomass yield formed a separate module that was linked to the other traits only through negative correlations with plant height and pod length, whereas pod width was linked only to seed weight per plant (r = 0.42). Because correlation does not establish causation, these associations are interpreted as co-variation of traits across treatments rather than as evidence that any individual trait determines yield.

## 3. Discussion

The main finding of this study is that shading had contrasting effects at the individual-plant and per-unit-area levels. Light shading (40%) increased pod weight, seed number and seed weight per plant by 25–31% on average, yet seed yield per hectare under 40% shading did not differ significantly from that under full sunlight, and biomass yield and seed yield decreased under 75% and 90% shading (Table 1 and Table 2). Moreover, the direction and magnitude of the responses depended strongly on variety and season, as shown by the significant Y × V × S interaction for all traits. The only response that was consistent across both varieties and both years was the reduction in seed yield under 90% shading (9–35%; Figure 9). Consequently, the 90% shading level, although it did not reduce all measured traits and even increased plant height and some per-plant yield components, was clearly detrimental to productivity per unit area.

Plant height increased under all shading levels, by 24–27% on average (Figure 1). Stem elongation under reduced irradiance is a typical shade-avoidance response through which plants increase internode length to place their leaves in better-lit positions [11,13], and variation in light intensity is known to affect plant height differently among species and varieties [28]. A similar increase in plant height under 70% shading was reported for okra grown under high temperatures [29]. In contrast, plant biomass increased under 40% and, to a lesser extent, 75% shading, whereas under 90% shading it did not differ from full sunlight on average and was lower than under full sunlight for Yaprak in 2022 (Figure 2). This suggests that 40% and 75% shading did not restrict assimilate supply at the individual-plant level, whereas 90% shading gave no further gain and, in some cases, limited dry matter accumulation, presumably because of reduced photosynthesis [24,30,31]. Taller plants with similar biomass under 90% shading indicate that resources were allocated to elongation rather than to dry matter accumulation. The different biomass responses of the two varieties are consistent with reports that shade tolerance varies among legume species and genotypes [14,22] and that genotype × environment interactions strongly influence legume performance [32].

Light shading (40%) increased the per-plant yield components, but this increase occurred in Araka in 2022 and in Yaprak in 2023 rather than in both varieties in both years (Figure 4, Figure 5, Figure 6 and Figure 7). A reduced radiation and heat load during flowering may limit the reduction in seed number, which in pea under high temperature has been attributed to flower and pod abortion [6,7,33] and, under high temperature and water deficit, to a lower plant growth rate during the reproductive period [27]. This interpretation agrees with the increase in pod weight per plant under low shading reported for faba bean [34], with the maintenance of flowering in okra under shading at high temperatures [29] and with the marked effect of shade intensity on the number of pods per plant reported for green gram [20]. The optimum for per-plant traits at 40% shading and their decline at higher shading levels resemble the response of cucumber, in which 35% shading increased fruit number whereas 60% shading reduced it [35], and indicate that above a certain shading level, the benefit of a reduced radiation load is outweighed by the reduction in photosynthetically active radiation [36,37,38]. Because the variety in which per-plant traits responded to light shading differed between years, the benefit of shading probably depends on the interaction between varietal development and the timing of seasonal stress. It should be emphasised that leaf and canopy temperature, soil moisture and incident radiation were not measured in the present study; the proposed reduction in heat load is therefore a hypothesis consistent with the data rather than a demonstrated mechanism.

Why did the gains in per-plant yield components not translate into higher seed yield per unit area? Within seasons, seed yield was closely related to biomass yield (r = 0.91) but not to any per-plant yield component (Figure 11), and the PCA separated the per-plant components (PC1) from the per-area yield traits (PC2) (Figure 10). This pattern indicates that differences in seed yield among treatments were determined mainly by stand-level biomass production rather than by the performance of individual plants. Two non-exclusive explanations are possible. First, seed yield per unit area is the product of the number of plants per unit area and seed yield per plant, and compensation among yield components is common in grain legumes; in faba bean, for example, seed size increased when shading severely reduced seed number [26]. Plants in less dense or less uniform stands can produce more pods and seeds per plant without increasing yield per unit area, so that individual plants sampled for the yield components may not represent the stand. Second, biomass yield, which integrates radiation interception and use by the whole canopy over the season, decreased under all shading levels (by 16–28% on average). Because seed yield depends on the amount of biomass produced [22,36], this reduction in canopy-level dry matter production offset the gains observed in individual plants. Plant density at harvest was not recorded. 

**Figure 12:**
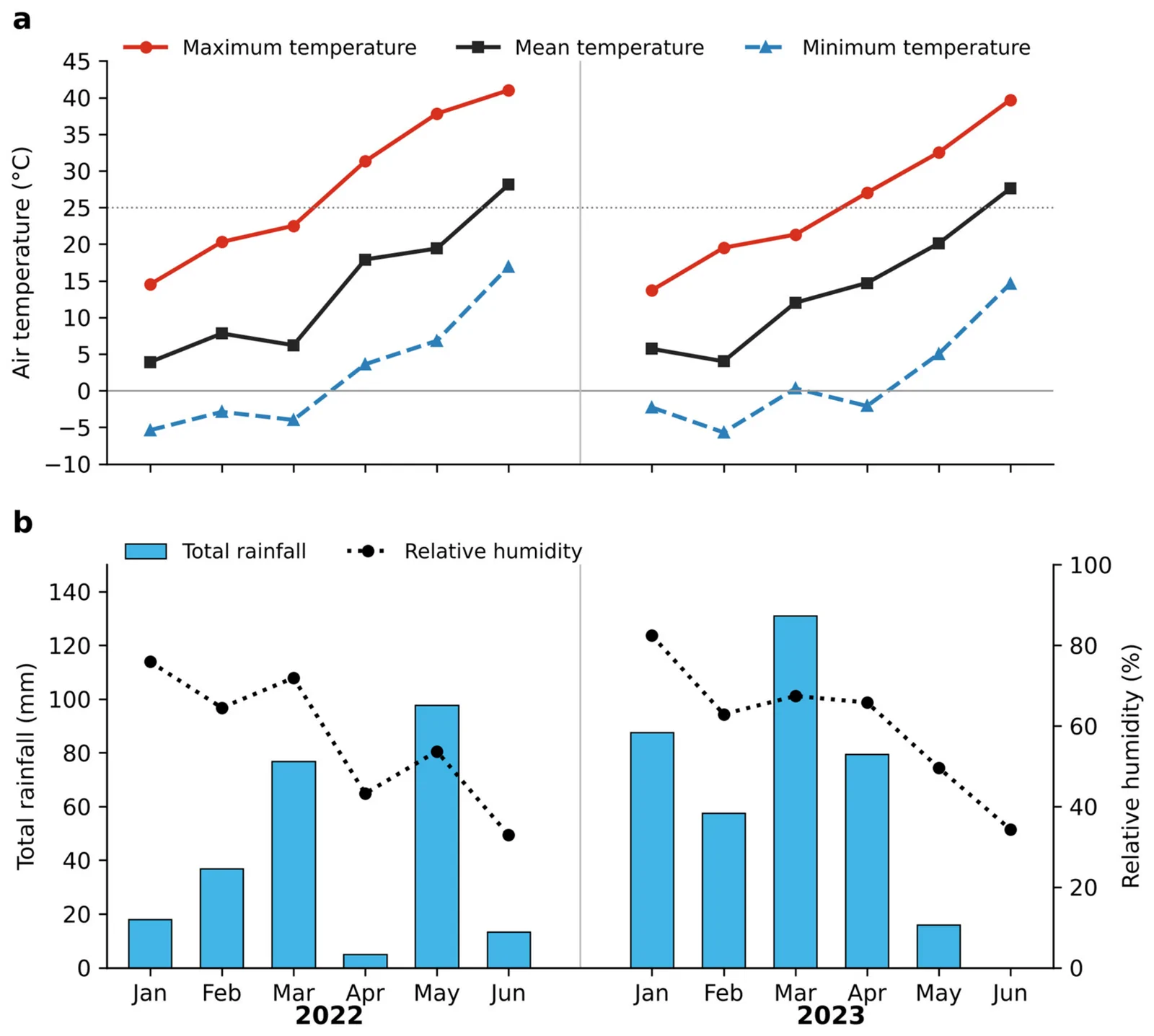
Monthly weather data recorded at the experimental site from January to June in 2022 and 2023. (**a**) Minimum, mean and maximum air temperature; the dotted line marks 25 °C. (**b**) Total rainfall (bars, left axis) and relative humidity (dotted line, right axis).

Why did Araka yield more under 75% shading than under full sunlight, whereas Yaprak yielded less? Araka produced 19–24% more seed under 75% shading than under full sunlight in both years, whereas Yaprak yielded 20–51% less under the same shading level (Figure 9). Yaprak produced more biomass and seed than Araka (2.92 vs. 2.13 t ha^−1^ and 1.24 vs. 0.75 t ha^−1^), particularly under full sunlight in 2023 (4.44 and 1.96 t ha^−1^), indicating that it was able to convert high irradiance into dry matter and seed efficiently; for such a genotype, reducing irradiance mainly limits the source of assimilates. Araka, which had a lower yield potential under full sunlight, may be more sensitive to the high radiation and temperature load of late spring, so that 75% shading alleviated a constraint rather than imposing one. Differences in shade tolerance among legume species have been reported [14,22] and are consistent with the strong V × S interaction for seed yield (Table 2). Nevertheless, the response of Yaprak also varied between seasons: its highest seed yield was obtained under 40% shading in 2022 and under full sunlight in 2023. The higher temperatures and lower rainfall during the reproductive period in 2022 compared with 2023 (Figure 12) may explain why light shading was beneficial only in the warmer, drier season, but this explanation needs to be confirmed by measurements of canopy temperature and soil moisture.

The large difference between the seasons also deserves attention. Seed yield was 46% higher in 2023 than in 2022, and the per-plant yield components were 2.2–3.4 times higher (Table 1 and Table 2). The 2023 season received 50% more rainfall from January to June and had lower maximum temperatures in May and June (Figure 12). Pea yield declines considerably when maximum daytime temperatures exceed about 25 °C during flowering [8,39], and high temperatures negatively affect pea yield under field conditions [33]; maximum temperatures above 30 °C were recorded in May in both years, i.e., during the reproductive period. These conditions confirm that heat and water deficit during the reproductive stage are major yield-limiting factors for rainfed pea in the region, which is likely to face higher temperatures and lower rainfall in the future [39]. Shading reduces incident radiation and has been shown to lower leaf temperature in mungbean grown under agrivoltaic panels [9], and in faba bean it prolonged seed filling and increased grain yield [23]. In the present study, shading did not increase seed yield on average, and its benefits were restricted to specific variety × season combinations.

The close intercorrelation among the per-plant yield components agrees with previous reports for pea [25,40], and multivariate approaches have been used in a similar way to interpret trait associations under environmental stress in chickpea and durum wheat [32,41]. However, the absence of a within-season correlation between these components and seed yield per unit area shows that evaluating shading responses on the basis of individual-plant traits alone may be misleading. The comparison of pooled and within-year correlations in this study also shows that pooling data from contrasting seasons without accounting for the year effect can produce spurious correlations, which should be avoided in multi-season shading studies.

## 4. Materials and Methods

### 4.1. Experimental Site Description

The field experiments were conducted during the early spring seasons of 2022 and 2023 at the research and trial area of the Department of Field Crops, Faculty of Agriculture, Dicle University, Diyarbakır, Türkiye (37°53′ N, 40°16′ E, 675 m above sea level). The mean annual temperature is 15.9 °C; the maximum daily temperature occurs in July and the minimum in January. The mean annual rainfall is about 495.9 mm. A harsh continental climate prevails and summers are hot and dry.

### 4.2. Soil Properties and Meteorological Conditions

The experimental soil was clay-loam textured with a pH of 8.15. It was low in organic matter, nitrogen and phosphorus, but rich in iron and calcium. The experiment was carried out under seasonal rainfall conditions. The meteorological base station located within the Field Crops Research Area of the University recorded weather parameters, including minimum, maximum and mean temperatures, relative humidity and rainfall, during the experiments. The meteorological data for the two years are given in Figure 12.

The records revealed that total rainfall at the experimental site in 2022 was 247.7 mm from January to June. In that year, rainfall was higher in March (76.9 mm) and May (97.8 mm) than in the other months, and only 5.0 mm fell in April. The maximum temperature reached 37.8 °C in May and 41.0 °C in June, and the mean temperature was 19.4 °C in May and 28.1 °C in June. The minimum temperature occurred in January (−5.4 °C) and March (−4.0 °C). The lowest relative humidity was recorded in June (32.9%), when evapotranspiration was intense (Figure 12).

Total rainfall was higher in the 2023 experimental season (371.5 mm) than in 2022 and was highest in March (131.0 mm). Maximum temperatures were lower in May (32.5 °C) and June (39.7 °C) than in 2022, whereas mean temperatures were similar (20.1 °C in May and 27.6 °C in June). The lowest minimum temperature of the 2023 season was recorded in February (−5.7 °C), and a late frost (−2.1 °C) occurred in April. Relative humidity was higher during the 2023 growing season than in 2022 and evapotranspiration was correspondingly lower (Figure 12).

### 4.3. Experimental Design and Treatments

The experiment was arranged as a split-plot design in randomized complete blocks with three replications in both years. Whilst two different pea genotypes were randomly allocated to the main plots, four different shading levels [full sunlight (control, 0 per cent), 40 per cent, 75 per cent and 90 per cent] were assigned to the sub-plots. The plant material was obtained from the Eastern Mediterranean Agricultural Research Institute, which operates under the General Directorate of Agricultural Research and Policies (TAGEM) of the Ministry of Agriculture and Forestry of the Republic of Türkiye. The ‘Araka’ variety (*Pisum sativum* L. cv. Araka) is widely preferred in Türkiye for both fresh consumption and the canning industry. The ‘Araka’ variety, which is a climbing type, is distinguished by its high chlorophyll content, large pods and the capacity to produce an average of 8–10 seeds per pod. Although it exhibits high tolerance to cool climatic conditions and early frosts during the vegetative growth stage, the ‘Araka’ variety is highly sensitive to biotic and abiotic stress factors, particularly water scarcity, during the generative stage (flowering and pod-filling stages). The ‘Yaprak’ variety (*Pisum sativum* L. var. *macrocarpon*) is entirely edible, including the pod wall; this variety differs from other peas in that the endocarp layer lacks sclerenchymatic fibrous tissue. Possessing a vigorous climbing growth habit and a robust vegetative structure, this variety, like other peas, prefers cool climatic conditions. However, due to its narrow harvest window, the pods in excessively hot regions tend to become fibrous, which leads to a loss of market value. The primary aim in selecting these genotypes is to comparatively evaluate the agronomic and morpho-physiological responses of pea materials, which possess different growth habits, plant architectures and pod morphologies to varying light conditions.

**Figure 13:**
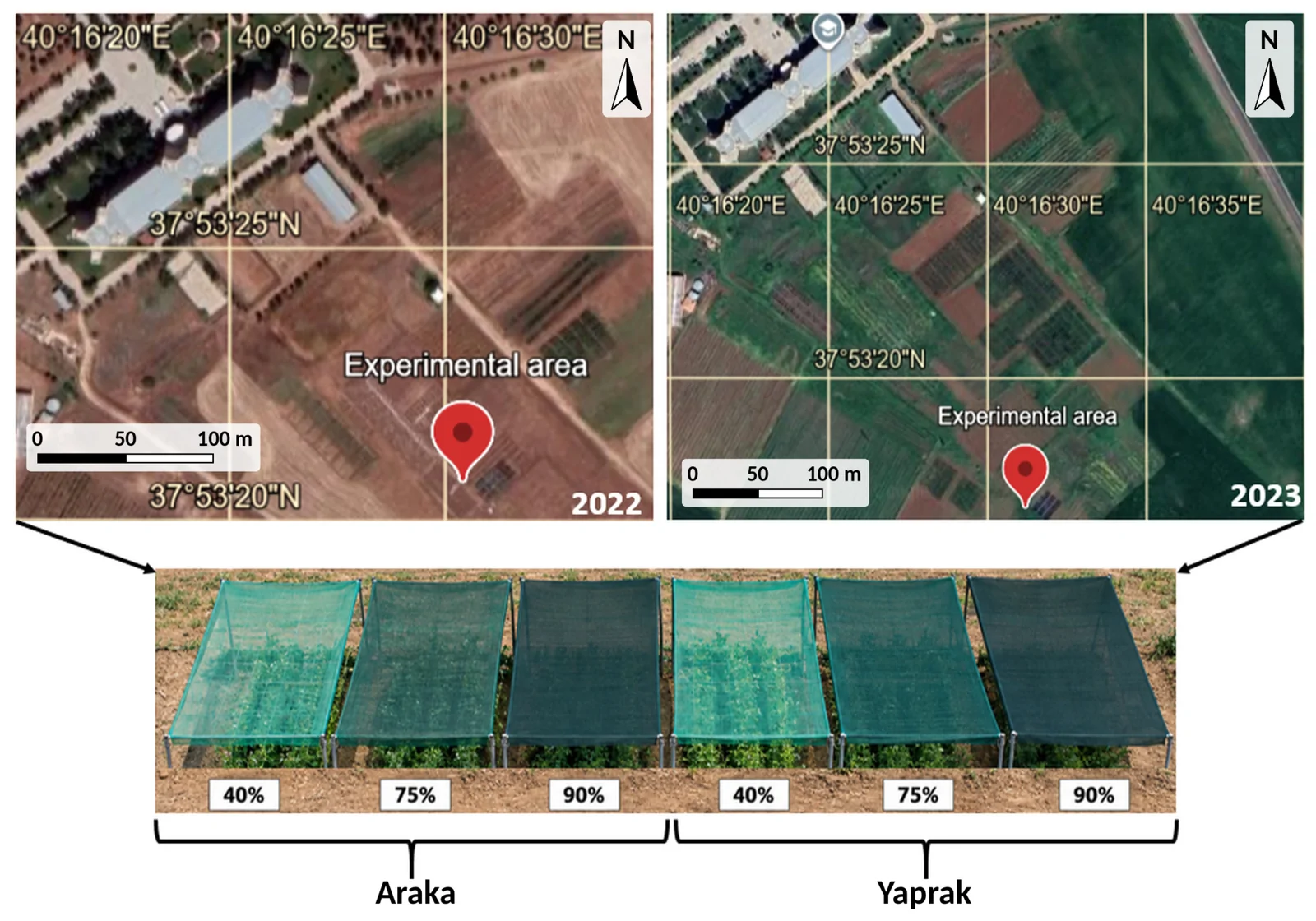
Experimental field areas in 2022 and 2023 and schematic illustration of the shade-net treatments applied to the two pea genotypes (Araka and Yaprak) under 40%, 75%, and 90% shading levels. The satellite images are shown with north at the top (north arrows), and the scale bar of each image was derived from its geographic coordinate grid.

Commercial shading nets with nominal shading capacities of 40%, 75%, and 90% were used in the shading treatments. These shading levels were selected because they represent commercially available net densities and allow for the assessment of plant responses to varying degrees of light restriction across a broad gradient. The 40% shading level represented a relatively low level of light restriction, the 75% level represented a high level, and the 90% level represented a very high level. Similar shading treatments have been employed in studies examining the effects of different plant densities on the yield and yield components of faba bean [34] and in research evaluating the performance of different plant species under shading structures [19]. Each subplot consisted of four rows, each 4.0 m in length with a row spacing of 0.45 m, resulting in a gross plot area of 7.2 m^2^. The seeding rate was standardized at 80 seeds m^−2^ for both genotypes. This seeding density corresponded to a seed quantity of 120 kg ha^−1^ for Yaprak and 176 kg ha^−1^ for Araka. Following plant establishment, an average of 65–70 plants were present in each 4 m row. To minimize edge effects, the two outer rows of each subplot were excluded from evaluation; observations, measurements, and harvesting were conducted on the two central rows, covering a net plot area of 3.6 m^2^. Seeds were sown on February 22 in both growing seasons. For the shading treatments, green, polyethylene-based, knitted commercial shading nets were used. The net shading capacity of the shade material was guaranteed by the manufacturer. As measurements of photosynthetically active radiation (PAR) or illuminance (lux) were not conducted during the study, the actual reduction in light occurring beneath the nets was not separately determined. Independent shading structures made of iron profiles measuring 2.0 m in height, 1.8 m in width, and 4.0 m in length were erected for each shaded subplot. A diagram of the experimental layout is given in Figure 13.

The shading nets were secured to cover only the top surface of the structures, leaving all side panels open. This arrangement aimed to maintain natural air circulation within the plots and mitigate the risk of heat accumulation often associated with enclosed shading systems. The shading structures were positioned with consideration for the sun’s path across the experimental site and were anchored to withstand precipitation and wind conditions. Separate structures were established for each replicate. No shading nets were used on the control plots. Shading treatments were initiated immediately after sowing in both growing seasons and continued until the plants reached physiological maturity. The experiment was conducted in the boundaries of the same research station during both growing seasons in different field areas. In the second year, the shading structures were re-established at the new trial site, and the varieties and shading treatments were re-randomized in accordance with the experimental design. This approach aimed to minimize spatial dependency potentially arising from the plot locations of the previous year. The plants were grown under natural rainfall conditions without irrigation, and weeds were controlled manually twice during the growing season. Plants were harvested by hand on 29 June in both years.

### 4.4. Measurements

Plant height, plant biomass, pod length, pod width, number of pods per plant, pod weight per plant, number of seeds per plant, and seed weight per plant were determined using 10 randomly selected plants from the two central rows of each subplot. All measurements were performed separately for each sampled plant, and the mean values of the ten plants were calculated to obtain a representative value for each subplot. Plant height (cm) was measured from the soil surface to the tip of the main stem. Plant biomass was determined as the total naturally dried aboveground biomass per plant, including stems, branches, leaves, pods, and seeds. The plants were maintained under the designated shading treatments until physiological maturity, after which the shade nets were removed and the plants were allowed to dry naturally under field conditions before harvesting. No oven-drying procedure was applied. Pod length (cm) and pod width (mm) were measured using a caliper on all pods collected from each of the ten sampled plants. The total number of pods and seeds per plant was recorded, and the pods and seeds were weighed separately to determine pod weight and seed weight per plant, respectively. The corresponding subplot means were calculated from the individual plant measurements. Biomass yield and seed yield were determined by harvesting the two central rows of each subplot, corresponding to a net harvested area of 3.6 m^2^. Ten randomly selected plants from these rows were used for individual plant measurements, and their biomass and seed weights were subsequently included in the total harvested biomass and seed yield of the corresponding subplot. Thus, all plants within the net harvested area were accounted for in the yield calculations. Biomass yield was determined from the total naturally dried aboveground plant material, including stems, branches, leaves, pods, and seeds, whereas seed yield was determined from the separated seeds. Seed weights were recorded at their naturally attained moisture content without adjustment to a standardized moisture level. The harvested biomass and seed weights from each net subplot area were converted to yield in t ha^−1^ using the following equation, in which multiplication by 10,000 converts kg m^−2^ to kg ha^−1^ and division by 1000 converts kg ha^−1^ to t ha^−1^:

Yield (t ha^−1^) = [net subplot weight (kg)/net harvested area (m^2^)] × 10,000/1000 (1)

### 4.5. Data Analysis

Data were analysed using a combined analysis of variance over the two years for a split-plot design in randomized complete blocks. Year was treated as a fixed effect because only two seasons were studied. The model included year (Y), replication within year (R(Y)), variety (V), Y × V, the main-plot error (R × V within year; error a), shading level (S), Y × S, V × S, Y × V × S and the subplot error (error b). Y was tested against R(Y), V and Y × V against error a, and S and all its interactions against error b. Normality of the residuals was checked with the Shapiro–Wilk test, and homogeneity of variances among the 16 Y × V × S combinations with Levene’s test. Variances were homogeneous for all traits (*p* > 0.18), and residuals were normally distributed (*p* > 0.13) for all traits except the number of seeds per plant and seed yield (*p* < 0.001 and *p* = 0.017, respectively). For these two traits, the analysis was repeated on log-transformed data, which gave normally distributed residuals and the same conclusions for S, Y × S and Y × V × S; untransformed means are presented. The analyses were performed in Python (version 3.12.7; Python Software Foundation, Wilmington, DE, USA) with the statsmodels (version 0.14.4) [42] and SciPy (version X1.14.1) [43] libraries; the mean squares were identical to those of the original analysis carried out with JMP Pro version 17 [44]. Means were compared using Fisher’s least significant difference (LSD) test at *p* ≤ 0.05, calculated from the error b mean square for comparisons among shading levels and V × S and Y × V × S combinations. Because the Y × V × S interaction was significant for all traits, means are presented for each year (Figure 1, Figure 2, Figure 3, Figure 4, Figure 5, Figure 6, Figure 7, Figure 8 and Figure 9), and means across years are given in Table 1 and Table 2. Error bars in Figure 1, Figure 2, Figure 3, Figure 4, Figure 5, Figure 6, Figure 7, Figure 8 and Figure 9 represent the standard error of the mean of the three replications of each Y × V × S combination; no data were pooled across years or varieties in the figures. Relative changes (%) caused by shading were calculated from the unrounded means as (treatment mean − full sunlight mean)/full sunlight mean × 100, i.e., the response ratio of the shaded treatment to the full sunlight control [45] expressed as a percentage change.

Principal component analysis and Pearson correlation analysis were performed on the 48 plot observations. Because the year effect was very large for most traits, each variable was standardised to zero mean and unit variance within each year before analysis, so that the multivariate structure reflected the variation due to variety, shading and replication rather than the difference between seasons; year was therefore not included as a variable. The suitability of the data for PCA was evaluated with the Kaiser–Meyer–Olkin (KMO) measure of sampling adequacy and Bartlett’s test of sphericity. Several traits were highly intercorrelated (Figure 11). Because PCA is computed from the correlation matrix and transforms correlated variables into uncorrelated components, collinearity does not bias the analysis; it is the reason for applying the method. However, highly correlated variables (e.g., the per-plant yield components) load on the same component and jointly increase its weight, and this was considered when interpreting the components. PCA was computed by eigendecomposition of the correlation matrix using NumPy [46], and loadings were calculated as eigenvectors multiplied by the square root of the corresponding eigenvalues. The contribution of each variable to a component was calculated as its squared eigenvector coefficient (%). Correlation coefficients were calculated between the within-year standardised variables, and their significance was tested with 45 degrees of freedom (n − 3, accounting for the removed year effect). Variables in the correlation matrix were ordered by hierarchical clustering (average linkage, distance 1 − |r|) using SciPy [43], and significant correlations (|r| ≥ 0.40, *p* < 0.01) were displayed as a network using the NetworkX library [47]. Data handling and visualisation were performed with pandas [48] and Matplotlib [49].

## 5. Conclusions

Under the rainfed semi-arid conditions of Diyarbakır, shading increased plant height and, at the 40% level, increased the per-plant yield components of field pea, but these gains did not translate into higher biomass or seed yield per unit area. Averaged over the two seasons and varieties, seed yield under 40% shading (1.09 t ha^−1^) did not differ significantly from that under full sunlight (1.10 t ha^−1^), whereas 75% and 90% shading reduced it by 13% and 25%, respectively, and 90% shading reduced seed yield of both varieties in both years. The response depended on variety and season: Yaprak gave its highest seed yield under full sunlight or 40% shading, whereas Araka produced more seed under 75% shading than under full sunlight in both years.

Therefore, 90% shading is not recommended for field pea. Under conditions similar to those of this study, light shading (40%) can be applied to high-yielding varieties such as Yaprak without a yield penalty, and may increase yield in hot, dry seasons; 75% shading may be considered only for lower-yielding varieties that, like Araka, responded positively to it in both seasons.

These recommendations are based on two contrasting seasons, two varieties and one location. The main limitations of the study are that leaf and canopy temperature, soil moisture, photosynthetic parameters and incident radiation under the nets were not measured, so the mechanisms proposed for the shading effects could not be tested directly, and that seed yield could not be decomposed into plant number and yield per plant. Future studies should include more varieties, seasons and locations, intermediate shading levels between 0% and 40%, shading applied only during the reproductive period, and measurements of microclimate, canopy temperature, soil water content and photosynthesis, together with an economic assessment of shade nets for field pea production.

## Figures and Tables

**Table 1:** Effect of shading level on plant height, plant biomass, pod length, pod width and number of pods per plant of two field pea varieties grown under rainfed conditions: variety × shading means across 2022 and 2023, main-effect means and analysis of variance. Year-specific means are shown in the figures of Section 2.1, Section 2.2, Section 2.3 and Section 2.4.

Variety	Shading Level	Plant Height (cm)	Plant Biomass (g Plant^−1^)	Pod Length (cm)	Pod Width (mm)	Pods per Plant
Araka	Full sunlight	58.50 d	15.28 c	13.31 a	18.56 ab	10.13 c
	40%	77.76 a	19.88 a	12.99 a	16.14 c	9.22 cd
	75%	72.17 b	15.96 c	11.50 b	14.19 d	7.97 e
	90%	76.96 a	15.89 c	10.90 c	16.47 c	9.93 c
Yaprak	Full sunlight	61.77 c	16.24 c	10.11 d	14.12 d	8.90 de
	40%	71.50 b	18.28 b	9.69 d	19.22 a	13.17 ab
	75%	76.66 a	18.22 b	10.70 c	17.61 bc	13.37 a
	90%	75.70 a	16.09 c	11.23 bc	11.17 e	12.27 b
2022		76.34 a	11.33 b	14.17 a	17.16 a	4.86 b
2023		66.42 b	22.63 a	8.44 b	14.71 b	16.38 a
Araka		71.35	16.75	12.18 a	16.34	9.31 b
Yaprak		71.40	17.21	10.43 b	15.53	11.93 a
Full sunlight		60.13 c	15.76 c	11.71 a	16.34 b	9.52 b
40%		74.63 b	19.08 a	11.34 ab	17.68 a	11.19 a
75%		74.41 b	17.09 b	11.10 b	15.90 b	10.67 a
90%		76.33 a	15.99 c	11.07 b	13.82 c	11.10 a
LSD (0.05)	V × S	2.39	1.28	0.58	1.48	0.97
	S	1.69	0.90	0.41	1.05	0.68
Source of variation	DF	MS	MS	MS	MS	MS
Y	1	1180.49 **	1533.79 **	394.63 **	72.28 **	1590.45 **
R(Y)	4	6.38	1.53	1.00	0.26	0.78
V	1	0.04 ns	2.44 ns	36.47 **	7.92 ns	81.90 **
Y × V	1	1058.36 **	43.26 **	111.52 **	7.13 ns	105.91 **
Error a	4	6.43	1.15	0.39	1.49	0.55
S	3	682.89 **	27.59 **	1.06 *	30.75 **	7.11 **
Y × S	3	395.44 **	7.56 **	4.74 **	5.44 *	10.75 **
V × S	3	71.63 **	7.78 **	9.79 **	66.44 **	24.43 **
Y × V × S	3	1169.01 **	35.89 **	16.56 **	8.24 **	27.20 **
Error b	24	4.01	1.15	0.24	1.54	0.66
CV (%) (error b)		2.80	6.31	4.29	7.79	7.65

Y, year; R(Y), replication within year; V, variety; S, shading level; error a, replication × variety within year (main-plot error); error b, subplot error. Y was tested against R(Y), V and Y × V against error a, and S and its interactions against error b. * and ** indicate significance at *p* ≤ 0.05 and *p* ≤ 0.01, respectively; ns, not significant. Within each group of means in a column (variety × shading, year, variety and shading level), means followed by the same lowercase letter do not differ significantly according to Fisher’s LSD test at *p* ≤ 0.05; year and variety means without letters do not differ significantly.

**Table 2:** Effect of shading level on pod weight per plant, number of seeds per plant, seed weight per plant, biomass yield and seed yield of two field pea varieties grown under rainfed conditions: variety × shading means across 2022 and 2023, main-effect means and analysis of variance. Year-specific means are shown in the figures of Section 2.5, Section 2.6, Section 2.7, Section 2.8 and Section 2.9.

Variety	Shading Level	Pod Weight per Plant (g)	Seeds per Plant	Seed Weight per Plant (g)	Biomass Yield (t ha^−1^)	Seed Yield (t ha^−1^)
Araka	Full sunlight	10.85 cd	46.62 c–e	8.14 cd	2.55 c	0.75 d
	40%	13.41 b	52.60 b–d	10.64 a	1.78 f	0.72 e
	75%	9.95 d	39.50 e	7.61 de	2.20 d	0.90 c
	90%	12.34 b	53.88 a–d	8.40 cd	1.97 e	0.63 f
Yaprak	Full sunlight	10.85 cd	43.43 de	8.12 cd	3.57 a	1.46 a
	40%	14.92 a	64.57 a	9.69 ab	3.38 b	1.46 a
	75%	12.05 bc	55.30 a–c	8.94 bc	2.20 d	1.01 b
	90%	12.49 b	58.73 ab	6.58 e	2.51 c	1.02 b
2022		7.57 b	24.07 b	5.30 b	2.18 b	0.81 b
2023		16.64 a	79.59 a	11.73 a	2.86 a	1.18 a
Araka		11.63	48.15 b	8.70	2.13 b	0.75 b
Yaprak		12.58	55.51 a	8.33	2.92 a	1.24 a
Full sunlight		10.85 c	45.02 b	8.13 b	3.06 a	1.10 a
40%		14.17 a	58.58 a	10.17 a	2.58 b	1.09 a
75%		11.00 c	47.40 b	8.27 b	2.20 d	0.96 b
90%		12.41 b	56.31 a	7.49 b	2.24 c	0.83 c
LSD (0.05)	V × S	1.40	10.92	1.26	0.055	0.022
	S	0.99	7.72	0.89	0.039	0.016
Source of variation	DF	MS	MS	MS	MS	MS
Y	1	986.56 **	36,996.31 **	497.46 **	5.5192 **	1.6530 **
R(Y)	4	4.09	74.65	0.31	0.0033	0.0000
V	1	10.78 ns	649.74 *	1.58 ns	7.4798 **	2.8283 **
Y × V	1	23.59 *	1584.70 **	0.01 ns	9.0784 **	1.8636 **
Error a	4	1.52	69.53	1.08	0.0024	0.0004
S	3	28.52 **	526.38 **	15.93 **	1.9283 **	0.2008 **
Y × S	3	15.02 **	445.61 **	2.06 ns	0.4771 **	0.0321 **
V × S	3	3.18 ns	209.92 ns	5.48 **	1.3918 **	0.2647 **
Y × V × S	3	34.58 **	758.97 **	14.45 **	1.6053 **	0.2507 **
Error b	24	1.38	83.93	1.13	0.0021	0.0003
CV (%) (error b)		9.71	17.68	12.46	1.83	1.86

Y, year; R(Y), replication within year; V, variety; S, shading level; error a, replication × variety within year (main-plot error); error b, subplot error. Y was tested against R(Y), V and Y × V against error a, and S and its interactions against error b. * and ** indicate significance at *p* ≤ 0.05 and *p* ≤ 0.01, respectively; ns, not significant. Within each group of means in a column (variety × shading, year, variety and shading level), means followed by the same lowercase letter do not differ significantly according to Fisher’s LSD test at *p* ≤ 0.05; year and variety means without letters do not differ significantly.

## Data Availability

The data presented in this study are available on request from the corresponding author.

## Abbreviations

The following abbreviations are used in this manuscript:

BY	Biomass yield
CV	Coefficient of variation
DF	Degrees of freedom
LSD	Least significant difference
MS	Mean square
NP	Number of pods per plant
NS	Number of seeds per plant
PB	Plant biomass
PC	Principal component
PCA	Principal component analysis
PH	Plant height
PL	Pod length
Pweight	Pod weight per plant
Pwidth	Pod width
SW	Seed weight per plant
SY	Seed yield
KMO	Kaiser–Meyer–Olkin measure
R(Y)	Replication within year
S	Shading level
V	Variety
Y	Year
